# Cardiac Sympathetic Denervation in Channelopathies

**DOI:** 10.3389/fcvm.2019.00027

**Published:** 2019-03-26

**Authors:** Veronica Dusi, Gaetano Maria De Ferrari, Luigi Pugliese, Peter J. Schwartz

**Affiliations:** ^1^Department of Molecular Medicine, Section of Cardiology, University of Pavia, Pavia, Italy; ^2^Cardiac Intensive Care Unit, Arrhythmia and Electrophysiology and Experimental Cardiology, Fondazione IRCCS Policlinico San Matteo, Pavia, Italy; ^3^Unit of General Surgery 2, Department of Surgery, Fondazione IRCCS Policlinico San Matteo, Pavia, Italy; ^4^Center for Cardiac Arrhythmias of Genetic Origin and Laboratory of Cardiovascular Genetics, Istituto Auxologico Italiano, IRCCS, Milan, Italy

**Keywords:** sudden cardiac death, cardiac sympathetic denervation, long QT syndrome, catecholaminergic polymorphic ventricular tachycardia, cardiac autonomic nervous system

## Abstract

Left cardiac sympathetic denervation (LCSD) is a surgical antiadrenergic intervention with a strong antiarrhythmic effect, supported by preclinical as well as clinical data. The mechanism of action of LCSD in structurally normal hearts with increased arrhythmic susceptibility (such as those of patients with channelopathies) is not limited to the antagonism of acute catecholamines release in the heart. LCSD also conveys a strong anti-fibrillatory action that was first demonstrated over 40 years ago and provides the rationale for its use in almost any cardiac condition at increased risk of ventricular fibrillation. The molecular mechanisms involved in the final antiarrhythmic effect of LCSD turned out to be much broader than anticipated. Beside the vagotonic effect at different levels of the neuraxis, other new mechanisms have been recently proposed, such as the antagonism of neuronal remodeling, the antagonism of neuropeptide Y effects, and the correction of neuronal nitric oxide synthase (nNOS) imbalance. The beneficial effects of LCSD have never been associated with a detectable deterioration of cardiac performance. Finally, patients express a high degree of satisfaction with the procedure. In this review, we focus on the rationale, results and our personal approach to LCSD in patients with channelopathies such as long QT syndrome and catecholaminergic polymorphic ventricular tachycardia.

## Introduction

The management of patients at risk of life-threatening arrhythmias is challenging, more now than ever. On one hand, our capability to identify the subjects at higher risk of sudden cardiac death (SCD) is still limited (1). On the other, the widespread availability of implantable cardioverter defibrillators (ICDs) is a double edge sword. Not only because of the risk of side effects but also because in peculiar settings ICDs may even become pro-arrhythmic. Additionally, recurrent ICD shocks have a dramatic impact on the quality of life. These drawbacks are particularly evident in young patients with inherited arrhythmogenic disorders. The management of these subjects is further complicated by the unlikely feasibility of randomized clinical trials in this setting, which may give the wrong perception of lack of strong evidence for a specific treatment. Left cardiac sympathetic denervation (LCSD) is an extremely effective but still underutilized anti-adrenergic therapy. LCSD has a strong physiological rationale, combined with consistent preclinical results, and clinical data from well-conducted multicenter registries.

In this review we will first summarize the history and the antiarrhythmic rationale for LCSD, including well-established antiarrhythmic mechanisms as well as potential new mechanisms. Then, we will present the clinical results of LCSD in Long QT Syndrome (LQTS) and Catecholaminergic Polymorphic Ventricular Tachycardia (CPVT), including both secondary and primary prevention. Finally, we will provide our approach for LCSD use in LQTS and CPVT.

## Organization of Cardiac Sympathetic Nervous System in Humans

**Figure 1:**
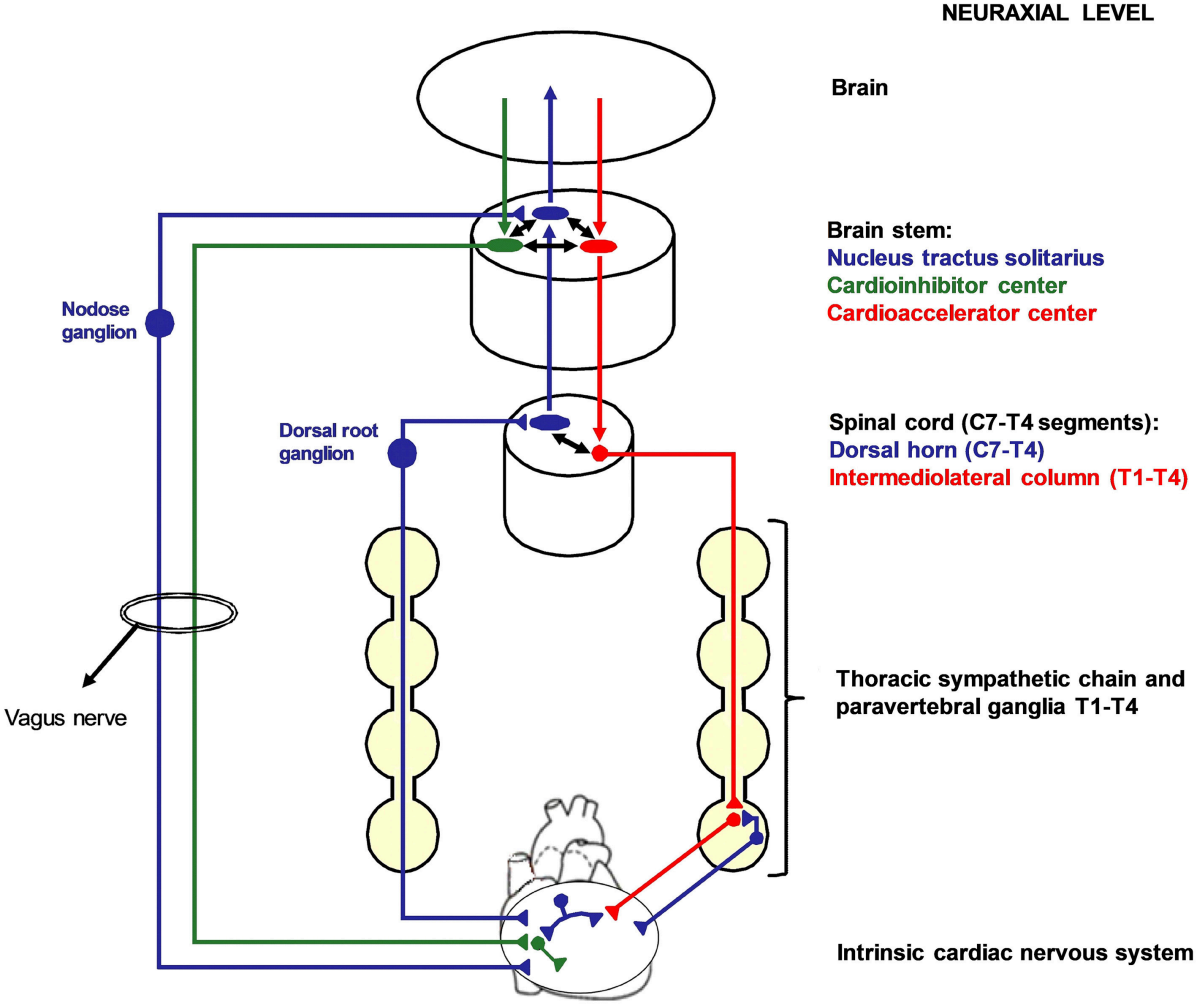
Cardiac nervous system organization in humans. Blue: afferent nervous system with its ganglia: nodose ganglia and C7-T4 dorsal root ganglia (DRG). Green: parasympathetic efferent nervous system. Red: sympathetic efferent nervous system. All the afferent and efferent structures outside the central nervous systems are bilateral, although mostly represented as unilateral for simplicity. Cardiac afferent fibers traveling across the paravertebral sympathetic ganglia (usually referred to as cardiac sympathetic afferent fibers) directly reach the DRG without having synapsis before. These fibers mediate cardio-cardiac sympathoexcitatory spinal reflexes that significantly increase the sympathetic output to the heart. Left cardiac sympathetic denervation (LCSD) consists in the removal of the left thoracic sympathetic chain and paravertebral ganglia from T1 to T4. Since ipsilateral DRG are spared by LCSD, a left afferent reinnervation from the DRG to the heart is theoretically possible with time. On the other hand, the left efferent sympathetic system from T1 to T4 is interrupted at a preganglionic level; therefore, no ipsilateral efferent sympathetic reinnervation is possible after LCSD.

The two opposite branches of cardiac autonomic nervous system (ANS), namely the sympathetic and the parasympathetic nervous system, share a common embryological origin from the neuronal crest (2). The sympathetic cardiac ANS follows typical patterns in most people, although variants are seen (3, 4). It is constituted by the mediastinal cardiac plexus, the paravertebral sympathetic ganglia, the dorsal root ganglia (DRG), the spinal cord, and the brain stem. Cardiac sympathetic afferent fibers provide beat-to-beat information centrally as their sensory endings are mechanoreceptors (5). The extracardiac afferent stations, containing pseudounipolar nerve cells, are the DRG from C7 to T4 spinal cord level. Of note, cardiac sympathetic afferent fibers travel across the paravertebral sympathetic ganglia (without having synapsis) before reaching the DRG. Efferent sympathetic preganglionic neurons have their soma in the intermediolateral column of spinal cord and synapses on postganglionic neurons located in the lower cervical and upper thoracic paravertebral ganglia. The lowest cervical ganglion (C8) and the highest thoracic ganglion (T1) are generally fused bilaterally to constitute the left and the right stellate ganglia (also referred to as cervicothoracic ganglia). In <3% of human sympathetic chains, the second thoracic ganglion (T2) is fused as well, constituting a trilobal (C8-T1-T2) stellate ganglion (3). The stellate ganglia convey a consistent amount of cardiac sympathetic postganglionic fibers. The remaining is provided by T2–4 paravertebral ganglia. Figure 1 summarizes cardiac nervous system organization in humans.

## Historical Prospective

In 1899, (6) Francois-Frank was the first to suggest that the removal of cervicothoracic sympathetic nervous system could prevent angina pectoris episodes. The first intervention was performed in 1916 by Jonnesco (7). He removed the left stellate ganglion (LSG) in a patient suffering incapacitating angina associated with cardiac arrhythmias, with effective and long-lasting suppression of both conditions. This pioneering intervention was strongly criticized due to the potential detrimental effects of depriving patients of the warning signal represented by pain. Moreover, the consequences of left stellectomy on coronary flow were still unclear. In 1929, Leriche and Fontaine (8) demonstrated that the sympathetic nerves exert a vasoconstrictive effect on the coronary arteries and not a vasodilator one, as previously thought. Subsequently, several clinical studies were performed in both Europe and the USA, confirming that left stellectomy was able to prevent anginal attacks (9), and to improve exercise tolerance (10). Concerning the optimal extension of the procedure, cervico-thoracic denervation (removal of the stellate ganglion and T2–T4 thoracic ganglia) proved to be the most effective. Finally, in the 60s, despite its clear efficacy, left cardiac sympathetic denervation (LCSD) was progressively abandoned for the treatment of angina due to the widespread usage of surgical coronary artery bypass graft and β-adrenergic-receptor blockers (11).

Except for some case reports (12, 13) the antiarrhythmic potential of cardiac sympathetic denervation in humans remained largely unexplored until the 70s. In 1971, Moss and McDonald (14) were the first to report LCSD in a LQTS patient with recurrent syncopal episodes. The rationale was based on a canine study (15) showing a consistent QT interval prolongation after either right stellate ganglionectomy or LSG stimulation. The patient underwent removal of the sympathetic ganglia from C7 to T2, including the entire LSG. Besides the suppression of arrhythmias, a persistent QT interval reduction was noticed. Subsequently, other groups tried to reproduce the beneficial effects on QT interval through the reversible percutaneous block of the LSG, with inconsistent results (16). Of note, at that time the appearance of Horner syndrome was considered as a good marker of the effective blockade of cardiac nerves. On the contrary, as pointed out already in 1975 (17), the Horner syndrome simply indicates an effective blockade of the sympathetic fibers traveling in the upper part of the stellate ganglion and innervating the eye. It does not necessarily indicate the block of the sympathetic fibers reaching the heart. Moreover, unlike in dogs and cats, in humans cardiac sympathetic innervation is not entirely provided by the stellate ganglia.

A better understanding of the rationale for LCSD in LQTS originated from the work by Schwartz and associates. Schwartz started from the observation in his first LQTS patient that sympathetic activation was triggering macroscopic T wave alternans, and he then reproduced in cats both QT prolongation and T-wave alternans by electrical stimulation of the LSG (18). On this basis, the young patient was treated with LCSD (remaining asymptomatic more than 40 years after) and sympathetic imbalance with left-sided dominance was proposed as the pathophysiological mechanisms of LQTS (17, 19). This concept prompted a large series of experimental studies investigating the consequences of unilateral (right or left) cardiac sympathetic denervation (20–22).

## Antiarrhythmic Rationale and Mechanisms of Action of Cardiac Sympathetic Denervation

### Antiadrenergic Effects

In 1976, Schwartz et al. showed in anesthetized dogs (20) that ischemia-related arrhythmias were increased by right stellate ganglion block and decreased by LSG block. In a conscious canine model (21), still considered as the most-clinically relevant experimental model of SCD (dogs with a healed myocardial infarction (MI) exposed to a brief coronary artery occlusion while exercising on a treadmill) left stellectomy confirmed its protective effect. The antagonism of ischemia-induced sympathetic activation (23) as well as the quantitative dominance of the left sided sympathetic nerves over the right (22) were the first antiarrhythmic mechanisms proposed for the protection associated with LSG block or removal. Next came the demonstration that VF threshold, a reliable and quantitative marker of cardiac electrical stability, was lower after unilateral right stellectomy and much higher after left stellectomy (24). These animal data provide a solid rationale for LCSD which goes far beyond LQTS and ischemia-related arrhythmias and could extend to every cardiac condition characterized by an increased susceptibility to VF. A major mechanism contributing to the protection is the net decrease in norepinephrine (NE) released in the left ventricle during sympathetic neural activation. Of note, the neural release of NE is an extremely inhomogeneous phenomenon (25–27). Indeed, sympathetic nerve stimulation rather than circulating norepinephrine, modulates T-peak to T-end interval (an ECG marker of dispersion of repolarization) by increasing global dispersion of repolarization (25, 28). In turn, a spatially inhomogeneous ventricular repolarization is a very well-defined pro-arrhythmic marker, both for scar related arrhythmias (29) and for functional reentrant arrhythmias such as polymorphic ventricular tachycardia and VF (30). The temporal dispersion of ventricular repolarization is important as well and, together with spatial dispersion, may lead to T wave alternans, an ECG marker of high electrical instability, both in case of macro- (18) and of microvolt alternans (31). Besides acting on the arrhythmic substrate, NE, like epinephrine, also modulates the trigger. Not only does it enhance automaticity in pacemaker cells in both the atria and the ventricles (32), but it also increases triggered activity including both early (EAD) (33) and delayed (DAD) afterdepolarizations (34, 35). Finally, LCSD has α-adrenergic-receptor blocking properties. Indeed, similarly to the effect of α-adrenergic-receptor blockade, and opposite to that of β-Blockade, LCSD increases myocardial reactive hyperemia, an index of the capability of the coronary bed to dilate (36). In addition to providing the basis for the antianginal effect, this could contribute to the antiarrhythmic efficacy (37).

### Vagotonic Effect

Animal studies clearly showed that LCSD is accompanied by a reflex increase in cardiac parasympathetic (vagal) efferent activity (38). In fact, LCSD interrupts the majority of centrally projecting cardiac sympathetic afferents, which have an inhibitory effect on the vagal outflow directed to the heart. In turn, experimental (39, 40) and clinical data (41, 42) showed that blunted vagal tone and reflexes can favor life-threatening arrhythmias; conversely, these arrhythmias can be counteracted in animals by direct vagal stimulation (43) or pharmacological activation (44). So far, the experience with direct vagal stimulation in humans is limited to heart failure patients (45–48). Accordingly, the vagotonic effect of LCSD is particularly relevant from an anti-arrhythmic point of view in conditions characterized by a chronic and progressive increase in sympathetic tone and a parallel decrease in central parasympathetic drive, such as myocardial infarction (MI) and heart failure (49). Some of these concepts are at the basis of an ongoing clinical trial which examines the potential benefit associated with LCSD in patients with advanced heart failure (50).

### Other Antiarrhythmic Mechanisms

#### Antagonism of Neuronal Remodeling

In 2000, Cao et al. (51) demonstrated in dogs that an increased intra-cardiac sympathetic nerve regeneration (nerve sprouting), obtained by infusing nerve growth factor to the LSG, was associated with a greater susceptibility to spontaneous ventricular arrhythmias. Of note, an intracardiac neuronal remodeling including both denervation and nerve sprouting (52) may occur after any kind of myocardial injury (53). Several animal studies consistently showed the high arrhythmic susceptibility of the denervated myocardium (54, 55). Similarly, in patients with cardiomyopathy and an ejection fraction ≤35%, the degree of cardiac sympathetic denervation quantified either by cardiac iodine-123 metaiodobenzylguanidine (123I- MIBG) imaging (56) or by positron emission tomography with 11 C-meta-hydroxyephedrine (11C-HED PET) (57) was significantly associated with ventricular arrhythmic risk. The process of neuronal remodeling is not limited to the heart, involving also extracardiac structures such as the sympathetic thoracic ganglia and the DRG (58). Myocardial infarction in animal models, independently of the site, is associated with an increase in nerve density, neuronal size, and neuropeptide Y expression in both the left and right stellate ganglia (59, 60). The same remodeling was described in humans. In 2012, Ajijola et al. (61) reported a significant neuronal enlargement and an increased synaptic density in the LSG of patients with refractory ventricular arrhythmias and structural heart disease undergoing LCSD. A few years later the same group further enriched the description of the sympathetic ganglia in patients with cardiomyopathy and refractory ventricular arrhythmias undergoing cardiac sympathetic denervation (62) showing the presence of a remarkable inflammatory cells infiltration (CD3+ T cells and neutrophils), combined with neurochemical remodeling, oxidative stress, and satellite glial cell activation. Of note, among the 16 patients studied (mean 45 ± 15 years), 5 had no macroscopically clear myocardial scar at pre-operatory multimodal imaging. Almost no signs of local inflammation or neuronal remodeling were observed in the stellate ganglia used as controls, obtained from 8 organ donors (mean 28 ± 8 years) with normal hearts deceased either for traumatic reasons or by natural causes.

These findings raise the intriguing question about the potential primary role of sympathetic ganglia inflammation in triggering adrenergic related ventricular arrhythmias in structurally normal hearts. Rizzo et al. (63) found mild but distinct inflammatory infiltrates composed of CD3+ and CD8+ T cells and macrophages in the LSG of 12 LQTS/ CPVT patients (mean 23 ± 17 years). They were all heavily symptomatic patients who received LCSD in secondary prevention. The authors specifically searched for neurotropic viruses as a potential trigger for the immune cell infiltration, with negative findings. They proposed that T-cell–mediated cytotoxicity toward ganglion cells may prompt an increase in sympathetic efferent activity toward the heart, therefore acting as a trigger and/or an enhancer of electrical instability in patients already predisposed to arrhythmias, as it occurs in LQTS and CPVT patients. Of note, as pointed out by Moss et al. (64) in the editorial comments of the paper, all patients had either recurrent syncopal episodes or many ICD shocks before the ganglionectomy, although the time frame between the last events and LCSD was not provided by the authors. Syncopal events are associated with transient generalized hypoperfusion, while ICD shocks can damage the myocardium and the neuronal fibers (65). Therefore, the mild auto immune mediated ganglionic remodeling observed by Rizzo et al. could be the consequence rather than the cause of the arrhythmic episodes. Moreover, the stellate ganglia used as controls, obtained from 10 accidently deceased patients (mean 35 ± 18 years), showed signs of inflammatory activity with the same immunohistological pattern, albeit to a lesser extent. Finally, no specific data supporting an increased sympathetic neuronal activity, such as increased neuronal size, increased synaptic density or a neurochemical shift in adrenergic phenotype were provided, as opposed to the neuronal hypertrophy and adrenergic shift demonstrated by Ajijola et al. (61, 62) in the stellate ganglia of patients with cardiomyopathy (even without overt scar) and intractable ventricular arrhythmias.

When interpreting these results, it's important to remember that cardiac sympathetic ganglia are not routinely evaluated by pathologist in the postmortem examination. Therefore, histological findings from these tissues among sudden arrhythmic death victims are lacking. On the other hand, a direct and non-invasive anatomopathological assessment of cardiac sympathetic ganglia in living patients is challenging both with labeled positron emission tomography tracers and with magnetic resonance. Indirect information about ongoing extracardiac neuronal remodeling processes can be obtained through cardiac 123I-MIBG or 11C-HED PET images, which are by the way unable to distinguish between anatomical (related to a reduced fiber density) rather than purely functional neuronal fibers abnormalities. As a matter of fact, an abnormal 123I-MIBG cardiac scintigraphy as compared with healthy controls was reported in LQTS (66, 67) patients as well as in patients with idiopathic ventricular tachycardia and fibrillation (68).

Overall, the intriguing question about the potential pro arrhythmic role of sympathetic ganglia inflammatory processes in channelopathies is still largely unsolved and should be properly assessed by larger studies. Nevertheless, cardiac 123I-MIBG data seem to support the presence of primary sympathetic nervous system abnormalities in these patients.

#### Antagonism of Neuropeptide Y

Neurotramitters other than NE released by sympathetic efferent fibers are an area of intense research. Co-release mainly occurs during high-level neuronal stimulation (69). The most studied sympathetic co-transmitter is neuropeptide Y (NPY) that has a long biological half-life and can be measured in peripheral blood (70). NYP was shown to inhibit acetylcholine (ACh) release from cardiac vagal postganglionic nerves (71–74) through Y2 receptors activation (75). NPY may also act on Y1 receptors on ventricular cardiomyocytes, affecting their electrophysiological properties. Optical mapping experiments in rats showed that NPY steepens the action potential duration restitution curve (76). Moreover, in Langendorff-perfused rat hearts with intact innervation only the combination of Y1 receptor antagonist with metoprolol was able to fully prevent the fall in VF threshold produced by prolonged high-frequency stellate stimulation (76). Finally, NPY is also a potent vasoconstrictor (77). In man, several studies already reported that plasmatic NPY levels rise following acute coronary syndromes (78) and in heart failure, showing a positive correlation with severity of heart failure and 1 year mortality (79, 80).

#### Correction of nNOS Imbalance

An additional neurotransmitter, which has recently gained attention, is neuronal nitric oxide (nNO). Neuronal nitric oxide synthase (nNOS), together with its adaptor protein (CAPON, codified by the gene NOS1AP, nitric oxide synthase 1 adaptor protein), is located in both intrinsic cardiac vagal neurons and postganglionic sympathetic neurons of the stellate ganglia. It acts locally as an intrinsic neuromodulator i.e., it is not released in the synaptic space but it acts in the synaptic cleft via stimulation of soluble guanylate cyclase, to generate cGMP. In turn, this prompts opposite effects in parasympathetic and sympathetic neurons. In parasympathetic neurons it leads to an increased release of Ach (81, 82), while in sympathetic neurons it causes a reduction in NE release (83, 84). Animal studies using viral vectors showed that an increase in nNOS may reverse impaired vagal (85) and exaggerated sympathetic drive (86, 87) in the spontaneously hypertensive rat. Moreover, in guinea pig overexpression of nNOS increased acetylcholine release and was associated with a trend of improved survival following MI (88). Interestingly, genetic studies not only consistently correlated genetic variation in NOS1AP with QT-interval duration in the general population (89–92), but also demonstrated their association with the risk for sudden death in general population (93) and the risk of drug-induced QT prolongation and ventricular arrhythmia (94). Additionally, NOS1AP was proved to be a genetic modifier in LQTS, both in a founder LQT1 population (95) and in a non-selected LQTS population including different genotypes (96). Of note, NOS1AP gene is also expressed at the cardiac level, and CAPON overexpression in isolated guinea pig myocytes causes attenuation of L-type calcium current, a slight increase in rapid delayed rectifier current (*I*Kr), and a shortening of action potential (97). So far, an increase in L-type calcium current (which is also enhanced by sympathetic activation) due to CAPON under expression has been advocated as the main mechanism responsible for NOS1AP genetic variant impact on QT interval duration and arrhythmias susceptibility. Nevertheless, it is intriguing to speculate that in LQTS patients (as well as in the general population), even in absence of overt inflammatory changes within the stellate ganglia, CAPON under expression (on genetic bases) may lead to an increased NE release during sympathetic activation and therefore an increased arrhythmic risk. Of note, the disruption in CAPON expression in LQTS could also be the functional result of a mild ganglionitis rather than the cause of it, potentially contributing to explain the pro arrhythmic impact of the mild auto immune mediated ganglionitis described by Rizzo et al. (63).

## Additional Effects of Cardiac Sympathetic Denervation on the Heart

Catecholamines, besides the arrhythmogenic potential, physiologically modulate nearly all cardiac functions, including inotropy, chronotropy, dromotropy, and lusitropy. Therefore, before systematically proposing LCSD in man, several experimental studies were performed in order to exclude any potential detrimental effect on the heart. In conscious dogs with a healed MI performing a submaximal exercise stress test, left ventricular contractility (assessed by dP/dt max) was not affected by left stellectomy (36). Moreover, LCSD did not reduce resting heart rate (HR) or chronotropic competence during effort. On the contrary, HR increase during exercise was slightly (6%) greater after LCSD. This apparently paradoxical effect was thought to be related to a controlateral reflex increase in right stellate ganglion activity. In fact, due to the asymmetric distributions of sympathetic cardiac nerves, the sinus node is under a predominant right-sided sympathetic control (98). In the same animal model (36) the maximal increase in HR during exercise was, respectively, 19 and 26% lower as compared to baseline (intact innervation) after bilateral and right only stellectomy. Finally, albeit no specific data about AV conduction were provided, the mean maximal HR reached (around 250 bpm) during effort after left stellectomy strongly argues against a significant impact of left stellectomy on dromotropy during sinus rhythm and in physiological conditions of sympathetic activation. This finding was in agreement with previous studies which showed that sympathetic innervation to the atria and the AV node is provided by both right and left sympathetic chain (99). Accordingly, recent data from patients with paroxysmal atrial fibrillation (AF) show equivalent electrophysiological effects of right and left stellate ganglion block (SGB) on both atria: unilateral temporary SGB with lidocaine slightly prolongs atrial effective refractory period and consistently reduces AF inducibility and AF episodes duration (100).

Finally, a last concern was that LCSD could lead to post-denervation supersensitivity, a pro-arrhythmic condition characterized by increased sensitivity of the left ventricle to catecholamines after complete denervation. From a theoretical point of view this possibility appeared unlikely, because right-sided sympathetic nerves (preserved after LCSD) are known to contribute to left ventricular innervation (101, 102). Animal studies confirmed that catecholamine stores in the myocardium were not completely depleted after LCSD (103, 104). Moreover, unilateral left stellectomy did not increase either dP/dt max or the incidence of ventricular arrhythmias in response to intravenous norepinephrine (105). Of note, LCSD is a preganglionic denervation; therefore no ipsilateral sympathetic efferent reinnervation is possible.

## LCSD in Channelopathies

### LCSD in Long QT Syndrome: Reported Results

The first large-scale evaluation of LCSD efficacy in LQTS was published in 1991 (106). Among the 85 reported patients, 99% were symptomatic before surgery, including 60% who suffered at least one aborted cardiac arrest (ACA). After LCSD, symptomatic patients decreased from 99 to 45% (*P* < 0.0001), and the mean number of cardiac events/patient dropped from 22 to 1. Of note, there were no ICDs. Therefore, this report truly reflects the impact of LCSD on SCD: it occurred in 8% of this high-risk group during 6 years of mean follow-up. The largest series of LQTS patients undergoing LCSD was reported in 2004 (107). As in the previous study, 99% of the patients were symptomatic before surgery, including 48% with a previous ACA and 75% with recurrent syncope despite maximum-dose β-Blockers. The majority were female (69%), the median age at surgery was 17 years and the mean QTc was 543 ± 65 ms. The average follow-up periods pre and post-LCSD were 5 and 8 years, respectively. After LCSD, 46% of the patients remained asymptomatic, syncope occurred in 31%, ACA in 16%, and SCD in 7%. Mean yearly number of cardiac events/patient dropped by 91% (*P* < 0.001). Among the 5 patients with a preoperative ICD the median number shocks/patient decreased from 25 to 0. Of note, 51 patients (35%) were genotyped, including 18 LQT1, 15 LQT2, 8 LQT3 and 9 patients with Jervell and Lange-Nielsen syndrome (JLN). As expected, LCSD appeared to be more effective in LQT1 than in LQT2. Despite the very limited numbers, patients with LQT3 and JLN did not seem to have a worse outcome compared with LQT1 patients. Finally, after LCSD a clinically significant mean reduction of QTc interval (39 ms) was noticed. Neither a preoperative QTc value ≥500 ms nor a change <40 ms were associated with a higher risk of recurrences. On the other hand, the persistence of a QTc ≥ 500 ms within 6 months from surgery appeared to carry a significantly higher risk of future events.

Subsequently, a large program of LCSD in LQTS was started by Ackerman at the Mayo Clinic, with equally positive results (108). In 2013, he reported a specific analysis on predictors of recurrences after LCSD in LQTS (109). They studied 52 consecutive LQTS patients undergoing LCSD between 2005 and 2010 at Mayo Clinic (23 LQT1, 9 LTQ2, 4 LQT3, 9 carrying multiple mutations, 3 JLN, and 4 genotype negative). All the procedures were performed using the minimally invasive, video-assisted thoracoscopic technique (VATS), and the sympathetic chain was removed from T1 to T4. Mean age at surgery was 10 years, 54% were female and mean QTc pre LCSD was 528 ± 74 ms. Most of them (61%) had LCSD as primary prevention because of either high-risk conditions or β-Blocker intolerance. This is a significant difference with the two previously reported populations and reflects the growing confidence in the benefit of the procedure. Overall, 12 subjects suffered cardiac events after LCSD (mean follow-up 3.6 years). Among them, only 5 (10%) had no discernible reduction of the arrhythmic episodes (true non-responders). These 5 high risk patients, all heavily symptomatic before LCSD, included 3 LQT3 patients and 2 LQT1 patients with multiple mutations. All of them had a very early onset of the disease (4 at birth, one in the first year of life) with QTc values above 600 ms. On the contrary, none of the 12 patients who received LCSD for β-Blocker intolerance experienced events during follow up.

In the following years, other centers all over the word started to perform LCSD and to report their results, overall confirming the positive post-procedural outcomes (110–112). The majority were small case series, yet in 2015 Waddell-Smith et al. (113) reported about 40 LQTS patients treated with thoracoscopic LCSD in New Zealand. LCSD related side effects and the quality of life after LCSD were the main topics analyzed. Most patients were female (70%) and LQT1 (57%), 11 were LQT2, 1 LQT3 and 5 had a negative genetic test. Half of the patients were completely asymptomatic before the procedure, and only 2 (5%) had surgery because of recurrences on β- Blockers. The two main indications for LCSD were β-Blocker intolerance or contraindication (35% of the patients) and β-Blocker non-adherence (25%). Interestingly, 10% of the patients specifically requested the procedure to their cardiologists either to increase their sense of protection or because of their desire to perform high level sports. These data confirm the diffusion and the increase in confidence in the procedure. During a median follow up of 2.5 years only 2 patients (5%), including 1 JLN, had arrhythmic events (syncopal episodes). All patients reported high levels of postoperative satisfaction. Table 1 summarizes indications and results of the largest case series reported of LCSD in LQTS with at least 1 year of follow up.

**Table 1:** Largest case series reported of LCSD in LQTS (at least 10 patients with at least 1 year of follow up).

**References**	***N***	**% Primary prevention**	**ICD**	**Mean follow up**	Overall cardiac events^*^	**ACA/ICD therapies**	**SCD**	**Resection sparing T1**
Schwartz et al. (106)	85	1%	0%	6 years	45%	0%	8%	0%
Ouriel et al. (114)	10	10%	0%	1.3 years	10%	0%	10%	0%
Schwartz et al. (107)	147	1%	3%	8 years	54%	16%	7%	0%
Li et al. (110)	11	0%	0%	3 years	45%	0%	9%	100%
Collura et al. (108)	18	50%	56%	1.5 years	17%	17%	0%	0%
Bos et al. (109)	52	61%	31%	3.6 years	23%	nr	2%	0%
Hofferberth et al. (111)	13	8%	nr	3 years	38%	23%	0%	92%
Olde Nordkamp et al. (112)	12	8%	67%	2 years	50%	25%	8%	0%
Waddell-Smith et al. (113)	40	95%	nr	2.5 years^**^	5%	0%	0%	72%
Jang et al. (115)	14	57%	nr	2.5 years	7%	7%	0%	0%

* *Syncope, aborted cardiac arrest, sudden cardiac death*.

** *Median follow-up. ACA, aborted cardiac arrest; ICD, implantable cardioverter defibrillator; nr, not reported; SCD, sudden cardiac death. The study by Antiel et al. (116) was not included despite describing 41 LQTS patients who received LCSD because specific data about the arrhythmic burden pre-post LCSD in the subgroup of LQTS patients were not provided*.

### LCSD in Long QT Syndrome: Our Approach

LCSD is now a mainstay in the management of LQTS patients (117, 118). Most experts agree that whenever ICD shocks occur in LQTS patients on optimized medical therapy, LCSD should be offered. We believe that, considering the high impact of LCSD on quality of life in this setting, the procedure should be undertaken without delay after the first breakthrough ICD intervention. ICD recurrences can be very detrimental and may lead to depression and even to suicidal attempts, particularly in these adolescents already predisposed to both anxiety and depression because of the underlying disease (119, 120). Moreover, the acute proarrhythmic potential of ICD shocks due to pain perception, fear and subsequent increase in the sympathetic drive on the heart should never be neglected, as will be discussed in detail for CPVT patients. The management of subjects with a first syncopal episode occurring despite maximum tolerated dose β-Blocker therapy is more challenging. As a referral group with a long-standing experience in the treatment of LQTS patients, we advise caution before directly implanting an ICD in these cases. Instead, a careful clinical evaluation is needed. Due to its high efficacy and optimal tolerability, we believe that LCSD should be offered first, clearly explaining to the patients and their families that the procedure is not an alternative to ICD implantation (that may always be considered in a later stage) and that the overall risk of life-threatening events after LCSD is low, unless the patient shows characteristics of high risk. At the same time, the life-spanning risk of complications and psychological consequences related to ICD implantation in these young patients is high (121) and should be properly acknowledged during patient and family counseling. Overall, a proper patient-physician communication in this setting requires to offer LCSD as therapeutic option even if the center is not performing the procedure as an inside facility. Ignorance and/or omission may carry medicolegal implications for the physician (122). On the other hand, in the case of markers of high risk such as onset of the symptoms in the first year of life and/or the persistence of QTc values exceeding 550 ms after LCSD, an ICD could be considered immediately after LCSD. Another difficult issue is the management of patients who never suffered arrhythmic episodes on therapy (and even before) but with either high risk LQTS phenotype or β-blocker intolerance, which represents the so-called primary prevention. In these cases LCSD should be offered before ICD implantation, with the clear intention to serve as bridge to an ICD in the most severe cases. Of course, additional pharmacological strategies such as mexiletine, already proposed in 1995 (123) and now widely used (124–126) should be offered as well, according to the genotype and the specific mutation. Finally, an additional indication for LCSD in LQTS is β-Blocker non-compliance. Generally, patients and their families managed in referral centers are well-instructed about the importance of strictly adhering to the prescribed medical therapy. Nevertheless, young subjects, particularly adolescents, are challenging to manage and may refuse therapy. Since β-Blocker non-compliance is a very well-defined risk factor for arrhythmic events in LQTS (127), if suspected and not modifiable, this condition should prompt to consider LCSD as additional protective measure. Concerning the indication to right cardiac sympathetic denervation (RCSD) in LQTS, we reserve it for patients not responding to LCSD. We discourage RCSD or a direct bilateral cardiac sympathetic denervation (BCSD) in patients not carrying an ICD (or pacemaker) due to the potential pro-arrhythmic effect of the induced (and largely unpredictable) bradycardia, particularly in LQT2 and LQT3 patients.

### LCSD in Catecholaminergic Polymorphic Ventricular Tachycardia

**Figure 2:**
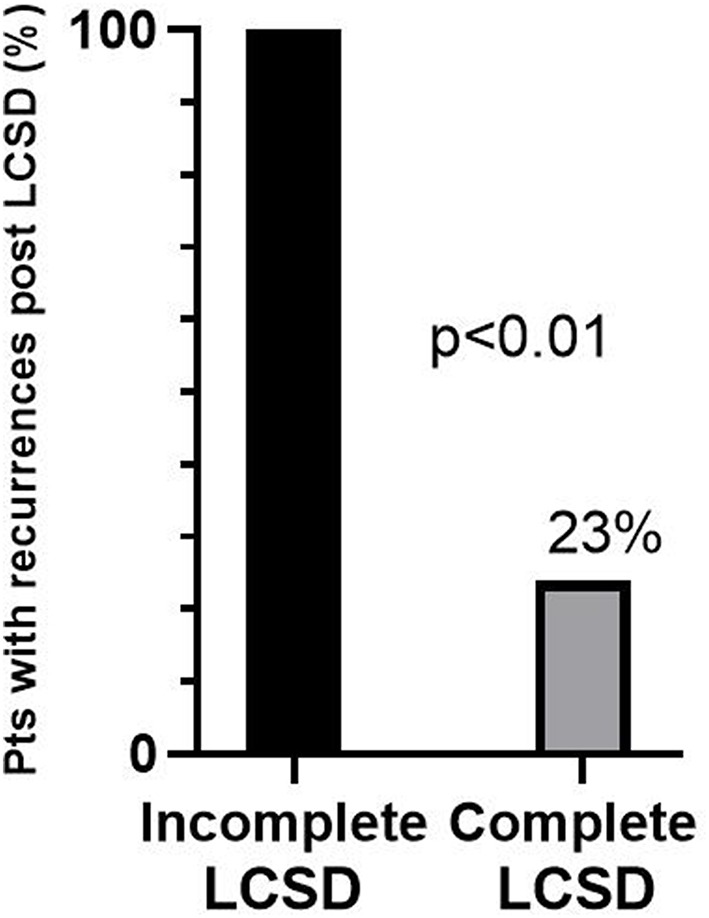
Percentages of recurrences after left cardiac sympathetic denervation (LCSD) in 38 CPVT patients who had previously suffered major cardiac events despite optimal medical therapy. The majority received a complete LCSD (*n* = 33), the remaining (*n* = 5) an incomplete LCSD. Modified from De Ferrari et al. (129) with permission.

The efficacy of LCSD in CPVT is not surprising from a pathophysiological point of view. Indeed, the disease is characterized by an intrinsic increase in the sensitivity of the heart to catecholamines due to mutations affecting the diastolic release of calcium from the sarcoplasmic reticulum. The first case series (3 patients) describing the long-lasting efficacy of LCSD in high risk CVPT was published in 2008 (128). We subsequently reported in 2015 the largest case series of LCSD in CPVT (129). It was a multicentric, international study involving 63 CPVT patients (71% RyR2 positive, 8% CASQ2 positive) who underwent LCSD between 1988 and 2014 at 11 centers worldwide. The majority (*n* = 54, 86%) had the procedure in secondary prevention, 97% were on β-Blockers, 24% on flecainide. The median post-LCSD follow-up was 37 months. In the 9 asymptomatic patients there were no cardiac events during follow-up. Among the 54 patients with prior major cardiac events either on (*n* = 38) or off (*n* = 16) optimal medical therapy, 13 (24%) had at least 1 recurrence, but only 1 patient died suddenly (after having been switched from nadolol to metoprolol). Specifically, the percentage of patients with cardiac events despite optimal medical therapy (*n* = 38) was reduced from 100 to 32% (*P* < 0.001) after LCSD, and among 29 patients with a pre-surgical ICD, the rate of shocks dropped by 93% from 3.6 to 0.6 per person per year (*P* < 0.001). Among the 13 patients with cardiac events after LCSD, only 5 (8%) had no reduction in the number of events as compared to before LCSD (true non-responders). Importantly, the only predictor of response was the extension of LCSD: 71% of the 7 patients with incomplete LCSD had recurrences as compared to 17% of those with a complete LCSD (*P* < 0.01). Among the 38 most severe patients, 100% of those with incomplete LCSD had recurrences (Figure 2). The most common reason for not performing a complete denervation was to reduce the risk of Horner syndrome. This is not justified since the incidence of permanent Horner syndrome when removing only the lower part of the stellate ganglion (T1) is extremely low (<2%). On the other hand, the antiarrhythmic protection when T1 is spared seems to be significantly lower, in agreement with pre-clinical data (130). In a subsequent exploratory sub analysis of the same population we focused on the 38 patients with an ICD (131). Our preliminary data suggest a reduction in supraventricular arrhythmias (SVA) leading to inappropriate ICD shocks after LCSD. Of course, this observation needs to be confirmed in a larger group of CPVT patients, but it seems very plausible from a pathophysiological point of view. Atrial arrhythmias (both atrial tachycardia and AF) in CPVT are typically triggered by catecholamines in the setting of structurally normal atria. Moreover, experimental animal models suggest that LCSD may increase the threshold for atrial arrhythmias onset and maintenance and reduce ventricular rate during atrial fibrillation (132–134).

Subsequently, a multicentric pediatric registry including 18 CPVT patients undergoing LCSD confirmed our results, showing no recurrences of ventricular arrhythmias in 89% of the subjects (135).

### LCSD in Catecholaminergic Polymorphic Ventricular Tachycardia: our Approach

LCSD is now an established therapy also for CPVT (117, 118). Our recommendations for LCSD in CPVT are similar to those already discussed for LQTS (first ICD shock or syncope on optimized medical therapy, β-Blockers intolerance or non-compliance), bearing in mind that the decision to implant an ICD in CPVT patients must be considered even more carefully than in LQTS. Indeed, due to the exquisite sensitiveness to catecholamine of their hearts, combined with a generally good hemodynamic tolerability of both rapid polymorphic VT and bidirectional tachycardia (which usually precede VF), CPVT patients are at high risk of electrical storms. This happens because the pain and the fear of the first ICD shock, which generally occurs in a condition of preserved consciousness, elicit a massive neural release of catecholamines, starting a vicious circle. As a matter of fact, in our registry of LCSD in CPVT (and therefore in an already selected subgroup of high-risk patients) we found that 36% of the patients who received an ICD before LCSD suffered at least one electrical storm or end of treatment condition (136). On the contrary, among the 26 pts with no ICD before LCSD, excluding two who had an electrical storm as first manifestation of the disease, none had such episodes on medical therapy. In agreement with this concept, sporadic cases of death in CPVT patients because of ongoing ventricular arrhythmias and exhaustion of ICD shocks have been reported for over 10 years (137–139). Very recently, the largest CPVT meta-analysis ever published (140) including 503 patients with an ICD (median age 15 years) reported a 1.4% mortality rate during follow-up, driven by 4 deaths due to electrical storms. The high incidence of both electrical storms (19.6%) and inappropriate shocks (20.8%) in trans venous ICD recipients is in full agreement with our data (129), as well as the disquieting rate of ICD-related complications (32.4%). Only 3 ICD patients had a subcutaneous ICD (S-ICD); 2 of them received inappropriate shocks due T-wave oversensing. Of note, the mortality rate among the 412 patients treated without ICD was similar to those with an ICD (2%).

Finally, beyond being potentially pro-arrhythmic and often not necessary, ICD shocks in CPVT patients may also be ineffective. Indeed, rapid polymorphic VT or bidirectional VT episodes may be not only self-limiting with the interruption of the stressor (such as physical activity) without the need for shock, but could also be less susceptible to cardioversion compared to VF episodes. Miyake et al. (141) demonstrated that among 10 CPVT patients who received a total of 75 appropriate shocks, only 57% of the shocks were successful in primary termination of the arrhythmias. The underlying rhythm in all successful ICD shocks at first attempt was VF, while no episode of polymorphic VT or bidirectional VT was successfully treated at the first attempt. Subsequently, Roses-Noguer F et al. (142) found an even lower success rate of the first appropriate ICD shock in CPVT (32%), confirming the ineffectiveness on triggered arrhythmias as compared to VF. Moreover, also antitachycardia pacing therapies (ATPs), as expected, proved to be ineffective in CPVT.

For all the above mentioned reasons, the management of high risk CPVT patients is particularly challenging. An optimized antiadrenergic therapy based on the clinical phenotype should always be the main therapeutic goal, whether or not the patient is implanted with an ICD (or is a candidate to). Indeed, in complete agreement with the pathophysiology of the disease, β-Blockers (143) and LCSD (129) are the only therapeutic interventions with a proven efficacy on SCD, aborted cardiac arrest and ICD shocks. Flecainide, despite promising *in vitro* (144) and *in vivo* (135, 144–147) data mainly showing its efficacy on effort induced arrhythmias, still lacks a validation on hard clinical end points. Nevertheless, a first pharmacological attempt with flecainide in association to β-adrenergic blockade seems reasonable in β-Blocker non-responders, particularly if the patient has already been implanted with an ICD. Finally, as for LQTS patients, a careful ICD programming with a single VF zone, long detection times and no ATPs, is crucial in CPVT patients.

## Conclusions

LCSD was proposed over one century ago for the treatment of angina pectoris. The antiarrhythmic potential of the technique, albeit evident since the first procedure by Jonnesco in 1916, took long to be fully appreciated (148). For many years the studies on LCSD were considered with skepticism, especially because there seemed to be just one group to support it. Finally, clinical data from well-conducted multicenter registries largely confirmed the preclinical findings, showing that LCSD is an effective treatment for drug-refractory ventricular arrhythmias in both LQTS and CPVT and LCSD is now recommended in recent guidelines (117, 118). Not surprisingly, considered the mechanism of action, the efficacy and potential indication of LCSD in channelopathies goes far beyond secondary prevention, potentially including many still asymptomatic patients with high-risk features for SCD despite optimized medical therapy. Regardless of this consistent body of evidence, LCSD is still an underutilized resource, as opposed to the often abused use of ICD in the same group of patients. From the technical point of view, the advantages of the thoracoscopic approach are such that it is difficult to see much room for different surgical approaches that might carry greater risks (149). LCSD can not only improve quality of life but also prevent fatal events that may still occur in patients with ICD due to the vicious circle of catecholamine-induced and maintained electrical storms.

## Author Contributions

VD and LP wrote a general manuscript draft. GD and PS supervised the process and edited the manuscript to its final version.

### Conflict of Interest Statement

The authors declare that the research was conducted in the absence of any commercial or financial relationships that could be construed as a potential conflict of interest.
